# Cytokine Pathways and Investigational Target Therapies in Hidradenitis Suppurativa

**DOI:** 10.3390/ijms21228436

**Published:** 2020-11-10

**Authors:** Ester Del Duca, Paola Morelli, Luigi Bennardo, Cosimo Di Raimondo, Steven Paul Nisticò

**Affiliations:** 1 Department of Health Science, University of Catanzaro Magna Graecia, 88100 Catanzaro, Italy; morellipaola1@gmail.com (P.M.); luigibennardo10@gmail.com (L.B.); steven.nistico@gmail.com (S.P.N.); 2 Department of Dermatology, University of Rome Tor Vergata, 00133 Rome, Italy; cosimodiraimondo@gmail.com

**Keywords:** hidradenitis suppurativa, cytokine, interleukin

## Abstract

Background: Hidradenitis suppurativa (HS) is a chronic inflammatory skin disease affecting areas with a high density of apocrine glands and characterized by subcutaneous nodules that may evolve into fistulas with pus secretion. Methods: The aim of this review is to investigate all current knowledge on cytokine regulation in the pathogenesis of HS. A systematic literature research using the words “cytokine”, “interleukin”, “pathway”, and “hidradenitis suppurativa” was performed in PubMed/Medline and Scopus/Embase databases. A search of the clinicaltrials.gov website for interventional recruiting and completed trials including the term “hidradenitis suppurativa” was also performed up to August 2020. We will discuss the pathogenetic role of various cytokines in HS and potential therapeutic targets for this debilitating disease. Results: The pathophysiology underlying this complex condition has not been clearly defined. An upregulation of various cytokines, such as tumor necrosis factor alpha (TNF-α), interleukin (IL)-1, IL-17, IL-23, and other molecules seems to be related to this inflammatory condition. Various cells, such as lymphocytes T Helper 1 and 17 and keratinocytes seem to be involved in the genesis of this condition. Conclusions: Several future studies and clinical trials are necessary in order to have new knowledge about HS and to properly treat this complex condition.

## 1. Introduction

Hidradenitis suppurativa (HS) is a chronic inflammatory condition primarily affecting apocrine-gland-rich regions of the body such as the axillary and groin areas [1,2]. HS presents with painful nodules and abscesses that may coalesce and form fistulas where the pus may drain. Lesions often evolve into scars, with a high physical and psychological impact for the patients [3]. Various therapies have been proposed to treat HS. Unfortunately, no therapy has been fully successful in the control of the disease [4,5,6,7,8,9,10]. Nowadays, the most effective treatment remains surgery [11]. To better define the possible treatments for this disorder, it is fundamental to assess the cytokine pathways involved in this condition, in order to develop new drugs that may lead to a better control of the disease. In this work, we are going to analyze all the possible cytokine pathways involved in the development of HS and which of these cytokines may be used as a possible target in the development of new drugs [12]. In this review, we aim discuss the pathogenetic role of various cytokines in HS and potential therapeutic targets attempted or currently under investigation for this debilitating disease.

## 2. Materials and Methods

**Figure 1:**
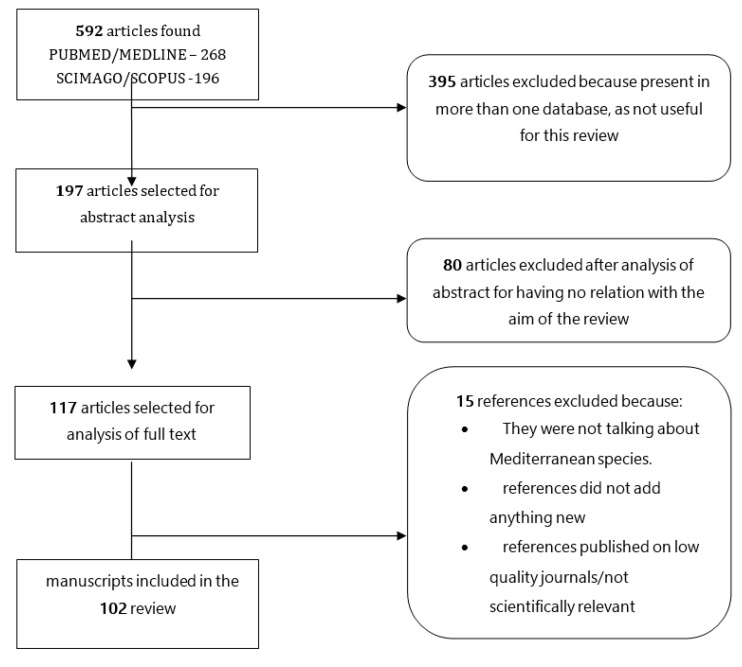
Article selection flowchart.

A systematic literature research was performed in PubMed/Medline, Scopus/Embase, and Google Scholar, in order to find articles suitable to be inserted in this review. Keywords used included “interleukin”, “cytokine”, “pathway”, and “hidradenitis suppurativa”. Duplicate articles were discarded before a full reading. Articles that did not bring any new information were excluded after a full reading. Article selection was performed independently by two researchers (L.B. and P.M.). Whenever discrepancies arose, a resolution was achieved by discussion with a third independent author (E.D.D.). The article selection flowchart is better described in Figure 1.

A search of the website clinicaltrials.gov for interventional recruiting and completed clinical trials with the term “hidradenitis suppurativa” was also performed up to 31 August 2020.

## 3. Results

A total of 197 non-duplicated citations were identified in the literature review (Figure 1). Eighty of these articles were removed upon review of titles and abstracts against the pre-defined eligibility criteria. Seventeen references were further excluded because they did not add any new information (Figure 1).

A total of 58 clinical trials were retrieved by a search of the clinicaltrials.gov database with the term “hidradenitis suppurativa” performed on 31 August 2020. Of these trials, 35 were completed and 23 were still recruiting patients. Twenty-seven completed and eight recruiting trials were utilizing immuno-modulatory treatments. Clinical trials’ characteristics are listed in Table 1 and Table 2.

### 3.1. Immunopathogenesis of Hidradenitis Suppurativa

HS pathogenesis is still largely unknown, and it is probably multifactorial [11]. Nevertheless, it is generally believed that follicular occlusion is the primary event, caused by hyperkeratinization and hyperplasia of the infundibular epithelium associated with defects in keratin production (downregulation of cytokeratin K17 and upregulation of K5 and K6) [12]. Genetics and lifestyle factors including smoking and obesity have been shown to contribute to the development of HS [47]. Follicular occlusion leads to dilatation of the hair follicle followed by rupture and release of contents, including hair-shafts, keratin fibers, microbes, and pathogen-/damage-associated molecular patterns (PAMPs/DAMPs), which leads to an acute and severe inflammatory response [48]. The release of the follicular contents into the dermis activates several inflammatory pathways, particularly (NOD)-like receptor protein 3 (NLRP3) inflammasome and toll-like receptor (TLR) signaling, thereby further aggravating the skin inflammation and the inflammatory loop [49]. Histologically, this event is characterized by cell infiltrates, including neutrophils (neutrophil elastase), T cells (CD3), B cells (CD19, CD20), plasma cells (CD138), natural killer (NK) cells (CD56), mast cells, macrophages (Factor XIIIA, CD68), and dendritic cells (CD11c, CD14). Multinucleate giant cells and body granulomas have also been identified in HS tissues [50]. Finally, as a specific hallmark of advanced HS, chronic inflammation induces sinus tract or tunnel formation. Studies of lesional tissue proposed the involvement of Ki67+ epithelial strands, the elevated proteolytic mechanism of metalloproteinases (MMP1, MMP2, and MMP8), and the increased activity of fibrotic factors such as transforming growth factor (TGF-ß 1-2-3) as fundamental in the sinus/tunnel formation [51,52,53]. These epithelialized cavities contribute to create a favorable habitat for biofilm-producing bacteria, which are able to trigger inflammation continuously [54].

### 3.2. Cytokines’ Role

**Figure 2:**
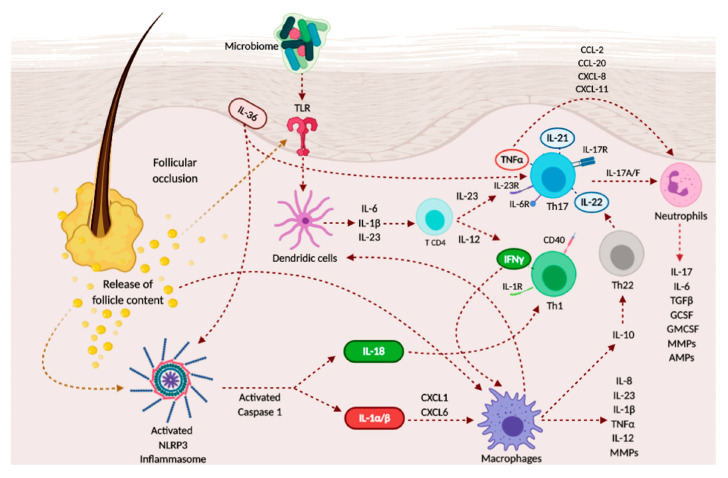
Schematic representation of the inflammatory pathways in hidradenitis suppurativa (HS), as for the pathways identified in this review.

Immune cells and keratinocyte-mediated products are widely accepted as key players in HS pathogenesis, and they appear dysregulated in lesional, perilesional, and normal-appearing tissue, serum and exudate of HS patients [55,56]. However, the exact role of each cytokine is not completely elucidated yet [55]. Cytokine-mediated keratinocyte hyperproliferation have been shown to contribute to the hyperkeratinization and hyperplasia of the infundibular epithelium in HS skin, leading to follicular occlusion with the subsequent formation of cysts [57]. Nonetheless, other trigger factors (e.g., mechanical friction) and/or predisposing factors (e.g., hair follicles of patients with HS seem to be susceptible to rupture due to alterations of the follicular structure) have been reported as contributory factors involved in the inflammatory loop of HS [58]. A representative scheme of the cytokines involved in the pathomechanism of HS is exemplified in Figure 2.

#### 3.2.1. Interleukin (IL)-1 Pathway 

It was proposed that the release of follicular content initiates the NLRP3 inflammasome, an innate immune signaling complex and key mediator of IL-1 family cytokine production. Upon activation, NLRP3 recruits the adapter molecule ASC (apoptosis-associated speck-like protein containing a caspase recruitment domain), which binds NLRP3 to pro-caspase-1. Caspase-1 is activated by autoproteolysis and formation of the enzymatically active heterotetramer. Active caspase-1 catalyzes the cleavage of inactive pro-IL-1β and pro-IL18 into active-form IL-1β and IL-18, respectively [59]. In several studies, elevated levels of caspase-1 with enhanced mRNA expression of NRLP3 were detected in HS lesions [60,61].

The IL-1 pathway is hyperactive and contributes to cell infiltration and tissue damage in HS. The IL-1 family consists of 11 members, 7 with a pro-inflammatory activity (IL-1α, IL-1β, IL-18, IL-33, IL-36α, IL-36β, and IL-36γ), while the remaining 4 have antagonistic (IL-1Ra, IL-36Ra, IL-38) [62] or anti-inflammatory (IL-37) [63] properties [64]. IL-1α is highly pro-inflammatory and induces a strong downstream of inflammatory cytokines such as tumor necrosis factor (TNF) and IL-18 [65,66].

IL-1β is produced mainly by monocytes and macrophages and contributes to amplify the inflammatory pathway leading to the induction of chemokines such as CXCL1 and CXCL6 involved in neutrophilic granulocytes’ recruitment [67]. Furthermore, IL-1β enhances the secretion of MMPs, which supports immune cell infiltration and contributes to tissue damage. Overexpression of IL-1β at mRNA and protein levels has been reported in lesional, perilesional, and normal-appearing HS skin compared to that in healthy control [60], and an active IL-1β pathway was also found in the serum of patients [68]. As result, IL-1β signaling induces overexpression of inflammatory markers, mainly IL-8, TNF-α, and IL-17 stimulating chemotaxis of new neutrophils into the damaged skin, pus formation, and triggering of the inflammatory loop [69].

Although the IL-1 pathway is well known to be activated in HS, Ardon et al. showed decreased levels of IL-1α in HS lesional skin compared with those in uninvolved skin of the same patient. Lower IL-1α levels in lesional HS skin may be related to the intracellular location and consumption of IL-1α at sites of inflammation [70]. Nonetheless, several data support that keratinocytes intrinsically produce increased levels of IL-1, thus it is supposed as a positive feedback between IL-1 and IL-17 [67]. In particular, IL-36, a member of IL-1 superfamily, is involved in the inflammasome activation and pro-inflammatory signaling through the activation of nuclear factor-kB (NF-kB) and mitogen-activated protein kinase (MAPK) [71]. In serum and lesional HS skin, several studies have proven increased levels of IL-36α, IL-36β, and IL-36γ and decreased antagonist cytokines (IL-36Ra, IL-37, IL-38) [72]. IL-36 increases dendritic cells activation, neutrophil recruitment, keratinocyte proliferation, and secretion of pro-inflammatory mediators (IL-1ß, TNF-α, IL-6, IL-8) stimulating the production of Th1 cells and Th17 cells and their cytokines (interferon gamma (IFN-γ), IL-17, IL-22, and IL-23) [73]. Regarding IL-36 antagonists, IL-37 and IL-38 levels have been significantly higher in perilesional HS skin than in healthy controls [72]. These cytokines are involved in the negative regulation of the inflammatory response, for example, by suppressing the secretion of the Th17 cells cytokines IL-17 and IL-22 [74,75]. The contributions of IL-37 and IL-38 imbalance in perilesional skin to the inflammatory pathogenesis of HS should be explored. IL-18 is secreted by macrophages and dendritic cells. Unlike IL-1β, there is a constitutive pool of pro-IL-18 in producer cells, thus the regulation of secretion is determined mainly by inflammasome activation. IL-18 promotes Th1 cell activation and increases the cytotoxic activity of CD8+ T cells and natural killer (NK) cells. In addition, IL-18 induces other inflammatory cytokines, especially IFN-γ. Typically, IL-18 activity is dramatically enhanced by other cytokines, including IL-2, IL-12, IL-15, IL-21, and IL-23. mRNA and protein expression of IL-18 showed high levels in lesional and perilesional HS skin [60,61]. As here depicted, the IL-1 pathway has been found upregulated in a large number of studies that investigated HS immune dysregulation, denoting a strong level of evidence for its role in HS pathogenesis [76].

#### 3.2.2. TNF-α and IFN-γ

TNF-α levels exhibited a positive correlation with HS severity and the therapeutic efficacy of TNF-α inhibitors supports the role of the dysregulated production of these cytokines in the pathophysiology of HS [77,78]. The only Food and drug administration-approved drug available to treat HS is adalimumab, a monoclonal antibody targeting TNF-α. As such, there is a high level of evidence on the upregulation of TNF-α in HS [79]. The TNF-α increases the ratio of Th17 to regulatory T-cells (Treg), resulting in aberrant production of Th17 cells and their cytokines IL-17 and IL-22 [80]. TNF-α in keratinocytes induces expression of CXCL8, CXCL11, CCL20, and CCL2 chemokines, which recruit neutrophils, T cells, and monocytes into the skin [81]. Together, these signals lead to massive immune cell infiltration into damaged tissue. Therefore, HS lesions are characterized by granulocytes, T cells, B cells, and monocytes, which differentiate into macrophages and dendritic cells. The dendritic cells mediators, IL-23 and IL-12, support Th17 and Th1 cells to produce their specific cytokines, IL-17 and IFN-γ, respectively.

IFN-γ is secreted by Th1 cells, induces Th1-attracting chemokines such as CXCL10, and activates dermal endothelial cells and macrophages allowing the infiltration of immune cells from the bloodstream [82]. IFN-γ mRNA and protein expression have been shown to be elevated compared to those in healthy controls in skin lesions and wound exudate, and its contribution has been assessed by several high-powered studies [77,83,84].

#### 3.2.3. IL-17/IL-23 Axis

Although it has been proven that Th1 cells plays an important role in the pathogenesis of chronic inflammatory conditions, several studies have found that Th17 exceeds the Th1 pathway in the induction of tissue inflammation [80,85,86]. Th17 cells are abundantly found in the papillary and reticular dermis of HS lesions and may be responsible for excessive neutrophilic inflammation and purulent drainage [86]. Th17 cell development is promoted by IL-23, IL-1β, and IL-6 produced by innate cells such as dendritic cells [87]. The Th17 axis encompasses several pro-inflammatory mediators such as IL-22, IL-21, IL-6, Granulocyte colony-stimulating factor (GCSF), IL-1β, TGFβ, and TNF-α and antimicrobial peptides (AMPs), in particular β-defensin-2, S100 proteins, and lipocalin-2 [88]. In keratinocytes, IL-17 induces the expression of LL37/cathelicidin, S100A7, S100A8, and S100A9, which are increased in the lesional tissue and serum of HS patients but not in perilesional skin [89,90]. These proteins are involved in keratinocyte proliferation and pro-inflammatory cytokine and chemokine expression. IL-17 overexpression has been detected in lesional, perilesional, and unaffected skin, suggesting that subclinical inflammation is present in HS skin prior to the formation of active lesions. Elevated IL-17 levels have also been registered in the serum of HS patients [91]. While the above-mentioned studies support a central role of Th17 cells in lesion development and driving HS inflammation, conflicting results were reported by one serum study, which showed no significant difference between patients and controls [92].

It has been reported that infiltrate macrophages in the papillary and reticular dermis of HS lesions overexpressed IL-23 [93]. Thus, considering the importance of the aberrant IL-17 expression, the IL-23/IL-17 axis is believed to be crucially involved in the pathogenesis of HS [86]. IL-23 is commonly expressed by macrophages in response to infectious stimuli and as mentioned above leads the differentiation of Th17 cells [94]. IL-23 is a member of the IL-12 family of cytokines, which also includes IL-12 and IL-27, and it is a heterodimer sharing a p40 subunit with IL-12 and having a distinct p19 subunit [95]. Increased mRNA expression of IL-23p19 in HS lesions and overexpression of IL-23p40 in serum has been proven [81,86]. IL-12 has also been observed in HS lesional skin in a limited number of studies. Remarkably, IL-12 and IL-23 cytokines are mainly produced by dendritic cells or macrophages and support the function of Th1 and Th17 cells, respectively forming their specific cytokines (IFN-γ, IL-17) [95].

#### 3.2.4. IL-6

IL-6 is a pleotropic cytokine that plays a key role in a wide variety of immune processes. IL-6 promotes antibody production by activated B cells, induces the expression of acute phase proteins such as C-reactive protein, and affects the function of several other cell types including keratinocytes. IL-6, in combination with TGF-β, IL-1β, and IL-23, promotes the development of Th17 cells and inhibits TGF-β-induced regulatory T-cell development [96]. At present, there are only a few evidences on the association between IL-6 and HS, but the results are controversial and the evidence is conflicting. Several studies show that IL-6 mRNA expression was increased in lesions of HS patients compared to that in non-lesional areas [56,97]. In contrast, other data revealed that the IL-6 levels in HS skin lesions were lower than those in non-lesional skin [98] and also that monocytes from HS patients exhibited in vitro an impaired ability to secrete IL-6 [55]. Elevated levels of IL-6 in the serum of HS patients have been described, and it has been suggested that IL-6 not only participates in the maintenance of inflammation but might also promote the formation of granulomas in lesions [99]. Notably, HS coexists with other inflammatory diseases where IL-6 also contributes to the development, including pyoderma gangrenosum and inflammatory bowel diseases, suggesting that they share similar immune–pathogenic pathways [99].

#### 3.2.5. IL-10

Unlike in other immune-mediated skin disorders, it has been observed that in HS there is a high expression not only of pro-inflammatory cytokines but also of the anti-inflammatory mediator IL-10 [84]. IL-10 is secreted by innate and adaptive immune cells, it induces the differentiation of Treg cells and suppresses the development of Th1, Th2, and Th17 cells [100]. IL-10 reduces immune responses by suppressing pro-inflammatory cytokine production by monocytes and macrophages and limiting T cell activation [101]. Several studies have demonstrated that the expression of IL-10 is elevated in HS lesional and perilesional skin [56,89,98]. Thus, the immunosuppressive role of IL-10 seems to be upregulated in HS skin as a compensatory response to the pro-inflammatory process and to the dissemination of skin commensal microbes. Moreover, this increase selectively suppresses not only IL-22 but also IL-17 lesional levels [84]. IL-22 is a member of the IL-10 cytokine family secreted by Th22 and other lymphocytic cells. It is known to promote keratinocyte hyperproliferation and epidermal acanthosis and to enhance AMP expression [102]. IL-22 has antimicrobial and pro-inflammatory functions and contributes to wound healing and the maintenance of epithelial barrier function [84]. The lack of IL-22 is associated with insufficient upregulation of AMPs, even in the presence of high levels of IL-17, which results in microbial spread in HS skin [103]. The decreased expression of IL-22 might be due to reduced infiltration of IL-22-secreting cells and impaired production of IL-22 by these cells [89,104]. IL-22 deficit has also been related to increase of IL-10 production, which might be induced, among others by IL-1β [84]. It is important to underline that IL-22 production is enhanced by Notch signaling, which is defective in HS patients [89]. On the other hand, some conflicting results have been found on the hyperexpression of the serum level of IL-10 in two studies that show no significant difference between the serum of HS patients and controls raising controversial ideas on the substantial role of this cytokine in the pathogenesis of HS [105,106]. Along with IL-10, other cytokines such as IL-4 and IL-13 have an anti-inflammatory role in HS. These cytokines can inhibit the synthesis of IL-1β, yet they stimulate the synthesis of IL-1Ra [64]. The specific role of these anti-inflammatory cytokines in the pathogenetic mechanism of HS remains to be further clarified.

### 3.3. Overview on Therapies and Treatment Possibilities

**Figure 3:**
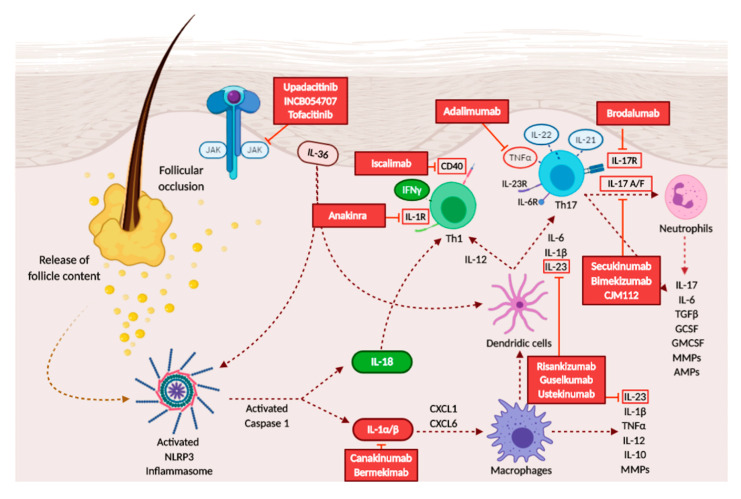
Schematic representation of the therapeutics targeting the immune products involved in HS pathogenesis.

Adalimumab, a monoclonal antibody directed against tumor necrosis factor-α, already approved for psoriasis and other various rheumatological and gastroenterological diseases [103], is the only biologic agent currently available for the treatment of moderate-to-severe HS resistant to antibiotics. However, several cases reported a certain rate of primary or secondary lack of response in some patients [107]. Numerous other specific anti-interleukins and small-molecules drugs are currently under investigation for the treatment of HS. The main HS-related drugs and their targets are represented in Figure 3.

A total of 21 biologics and other immunomodulatory agents reported in the treatment of HS were identified and categorized according to their mode of action and quality of evidence ranking as previously published [108] (Table 3). Among them, only the newest and the ones whose data are available on PubMed or on clinicaltrials.gov were discussed in this review, and levels of evidence have been added when available. We are providing below an up-to-date review of the most relevant clinical trials targeting the key products related to HS as shown in Table 1 and Table 2.

#### 3.3.1. TNF-α Inhibitors

● Adalimumab

Adalimumab is an IgG1 monoclonal antibody. In HS, it is administrated as an initial dose of 160 mg, followed by a dose of 80 mg for 2 weeks and a maintenance dose of 40 mg weekly. To date, adalimumab is the only FDA-approved biologic agent for the treatment of moderate/severe HS and a strong level of evidence, clinical trials, and case reports corroborate its use [109,110]. Two large double-blind, placebo-controlled, randomized clinical trials (RCTs), PIONEERI and II, demonstrated the safety and efficacy of adalimumab. In these studies, patients received either adalimumab (160 mg at week 0, 80 mg at week 2, and 40 mg weekly starting at week 4) or placebo for 12 weeks [78,110]. The primary endpoint in both studies was the number of patients achieving clinical response according to the Hidradenitis Suppurativa Clinical Response (HiSCR) at week 12 defined as a 50% decrease in total abscess and inflammatory nodule lesions from baseline. This endpoint was achieved by 41.8% and 58.9% of patients in the treatment groups versus 26.0% and 27.6% of patients in the placebo groups, for PIONEER I and II, respectively [110]. Despite providing clinical improvement, HiSCR is only achieved by approximately 50% of patients receiving adalimumab, and nearly 6.5% of patients developed anti-drug antibodies reducing drug efficacy [111].

● Infliximab

Infliximab (IFX) is a monoclonal antibody directed against TNF-α that inhibits its downstream effects [112]. A clinical trial on IFX dosed at 5 mg/kg at weeks 0, 2, and 6 followed by maintenance dosing every 8 weeks for 22 weeks, showed a decrease of HSSI >50% (HS-specific severity index) from baseline in 26.7% of patients [113,114]. Although a quite low number of patients met the primary endpoint, IFX undoubtedly presented an advantage over placebo in which the majority of patients (88.9%) showed less than 25% improvement in HSSI where severe disease is defined by an HSSI score ≥13 [114,115]. Despite the fact that further studies are needed to better define the use of IFX compared with that of adalimumab, IFX remains a valuable tool in the treatment of HS.

● Etanercept

Etanercept is a recombinant human TNF-α receptor that competitively binds TNF-α receptors [116]. Literature regarding the use of etanercept in HS has reported discordant data on its efficacy, and its current use in real life is limited [4,117,118].

#### 3.3.2. IL-1 Inhibitors

● Anakinra

Anakinra is a recombinant IL-1 receptor inhibitor [119]. In HS, it is subcutaneously administrated as a 100 mg daily dose [32,120,121]. A double-blind, randomized, clinical trial showed significantly decreased disease activity in the anakinra group compared to that in the placebo group at week 12 [121]. However, at 24 weeks, the difference in patients achieving HiSCR was not statistically significant (10% vs. 33%). Painful reactions at the injection site were commonly reported in rare cases also linked with drug-induced sarcoidosis [119,121]. Moreover, some reports showed anakinra failure in severe HS patients [122].

● Bermekimab

Bermekimab (MABp1) is an anti-IL-1α human monoclonal antibody [123]. Forty-two patients were enrolled in a phase 2 clinical trial, reporting that bermekimab was effectively inducing a clinical response after 12 weeks of treatment [123]. A significant reduction in abscesses and inflammatory nodules of 60% (*p* < 0.004) and 46% (*p* < 0.001) was seen in anti-TNF-naive and anti-TNF-failure groups, respectively. IL-1α could characterize an important clinical target for HS, and bermekimab may represent a new option to treat moderate-to-severe HS.

● MEDI8968 and Canakinumab

MEDI8968 [124] and canakinumab [125] are human monoclonal antibodies recently approved for rheumatologic conditions [124,126,127]. MEDI8968 selectively binds the IL-1R1 receptor inhibiting the activation of IL-1α and IL-1β, while canakinumab selectively targets IL-1β. A Phase IIa study was conducted to evaluate MEDI8968 for the treatment of moderate-to-severe HS patients, but it was terminated early due to a lack of efficacy [124]. Canakinumab showed mixed results in several case reports and series [128].

#### 3.3.3. Anti-IL-17 Drugs

Based on the key pathways involved in the pathogenesis of HS, several anti-IL-17 drugs are currently under investigation as possible efficacious therapeutics.

● Secukinumab

Secukinumab, an anti-IL-17A IgG1 antibody [103], has currently been studied in two Phase 3 double-blinded, randomized clinical trials (SUNSHINE and SUNRISE) [43,44]. It is given at the dosage of 300 mg subcutaneously per week, then followed by 4-weekly maintenance. Secukinumab was shown to improve HS condition in several case reports [129,130]. Phase 3 trials on secukinumab for treating HS are currently underway (Table 2), but results are still not available.

● Bimekizumab

Bimekizumab, an anti-IL-17A and anti-IL-17F IgG1 antibody, is under evaluation with two Phase 3 double-blinded, randomized clinical trials (BE HEARD 1 and 2) [17,42,103], with no results available at the time of writing. Among anti-IL-17 drugs, the ones that simultaneously block more subunits of anti-IL-17, such as Bimekizumab, may be more effective in the treatment of HS, as the various subunits seems equally involved in the development of inflammation, and bimekizumab seems to have a major effectiveness in suppressing inflammation and cytokine production from preclinical studies [103].

● Brodalumab

Brodalumab is a recombinant, fully human monoclonal antibody (IgG2), which binds with high affinity to the interleukin (IL)-17 receptor A (IL-17R). Brodalumab is FDA approved for the treatment of moderate-to-severe chronic plaque psoriasis [131].

A recently published study [85] reported promising results using brodalumab for the treatment of moderate-to-severe HS, along with no grade 2/3 adverse events. All patients in the study achieved HiSCR, and 80% achieved IHS4 (Severity Score System) [132] at week 12. HiSCR achievement occurred as early as week 2, likely due to the unique blockade of IL-17A, IL-17C, and IL-17F by brodalumab.

● CJM112

CJM112 is a human monoclonal anti-IL-17A antibody. A Phase II study with moderate-to-severe chronic HS has been completed, but results are not available at the present time [19].

#### 3.3.4. Anti-IL-23 Drugs

Two anti-IL-23 drugs, risankizumab [40,133] and guselkumab [22,134], are currently in phase 2 clinical trials to evaluate their efficacy in the treatment of moderate-to-severe HS. Various case series and isolated reports describe the effectiveness of this category of medication in the treatment of HS, as was reported for ustekinumab [103]. In future years, we expect the results of these trials that may bring a new weapon in the treatment of HS.

#### 3.3.5. Anti-IL-12/23 Drugs

● Ustekinumab

Ustekinumab is a human monoclonal antibody that acts by inhibiting the p40 subunit on IL-12 and IL-23 [127,135]. In a Phase II open-label study involving 17 patients, patients showed moderate-to-marked improvement achieving HiSCR in almost 40% of cases [136]. Several cases series reported positive outcomes using ustekinumab in moderate-to-severe HS patients [136,137,138].

#### 3.3.6. Janus Kinase (JAK) Inhibitors

● INCB054707

INCB054707 is an orally administered inhibitor of the JAK 1 pathway. There are currently two Phase II trials underway [15,16].

● Tofacitinib and Upadacitinib

Tofacitinib is a potent, selective JAK inhibitor that preferentially inhibits Janus kinase (JAK) 1 and JAK3 [139]. It has been recently shown to be potentially effective in recalcitrant HS by some case series serving as proof of concept for the ongoing clinical trial [46,140]. Upadacitinib is a selective JAK1 inhibitor, with 74- and 58-fold selectivity for JAK1 over JAK2 and JAK3, respectively [141]. A Phase 2, multicenter, randomized, double-blind study is currently recruiting moderate-to-severe patients to evaluate the safety and efficacy of this drug in treating HS [41].

#### 3.3.7. Others

● Apremilast

Apremilast is a small-molecule inhibiting phosphodiesterase 4 [142]. It blocks cyclic adenosine monophosphate (cAMP) degradation, which drives the activation of protein kinase A (PKA) and reduces production of TNF, IL-12p40, and IL-17 [143]. Apremilast has been tested in two double-blinded, phase 2 trials in patients with moderate HS. Eight out of 15 patients (53.3%) given apremilast achieved a positive HiSCR at week 16, compared to zero out of five patients in the placebo group (*p* = 0.055). Patients receiving apremilast also showed a lower rate of nodules and abscesses [144,145].

● IFX-1

IFX-1 is a human C5a-specific monoclonal antibody [31,146]. An open-label clinical trial reported that 75% of patients achieved HiSCR at day and more than 80% at day 134 [31]. No other information is available at the time of writing.

● Iscalimab (CFZ533)

Iscalimab CFZ533 is a fully human, Fc-silenced, non-depleting, IgG1 mAb preventing CD40 pathway signaling and activation of CD40+ cell types [147]. It is proposed for the treatment of several immune diseases [148,149]. A new study is currently ongoing to assess the preliminary efficacy and safety of CFZ533 in patients with moderate-to-severe HS to evaluate the clinical profile for further clinical development. A schematic representation of the main axes involved in the pathogenesis of HS and the most relevant drugs targeting those axes are shown in Figure 3.

### 3.4. The Role of Microbiome and Biofilms

The role of microbes in the pathogenesis of HS is still discussed. High levels of antimicrobial peptides including β-defensin-2, S100 proteins, lipocalin-2, and LL37/cathelicidin in HS skin [89] and the efficacy of antibiotics have suggested a strong microbial influence in disease activity [150]. However, it is unclear whether bacteria are initiating- and/or promoting-factors in the progression of HS or if sinus/tunnel formation provides a favorable habitat for biofilm-producing bacteria. Microbes probably trigger a cascade of PAMPs/DAMPs leading to NLRP3 activation and IL-1β release. In addition, TLR2 plays a central role in innate immunity, by sensing microbial ligands and activating the host defense response through inflammatory mechanisms. Recent findings of the highly increased expression of TLR2 by CD68+ macrophages and CD209+ in HS lesions suggest that microbial colonization might contribute to the tissue inflammation [77,151].

Culture-dependent studies of superficial and deep HS lesions have found the involvement of different microbial species [152]. Gram-positive cocci and rods, including *Staphylococcus aureus*, coagulase-negative Staphylococci (CoNSs), *Streptococcus* spp., and *Corynebacterium* spp. have been isolated from surface swabs and deep tissue samples [153,154]. CoNS species were the most common species found in bacterial cultures of deep HS lesions obtained by carbon dioxide laser treatment [155]. *Staphylococcus lugdunensis* and other CoNS species such as *Staphylococcus epidermidis* were associated with the early stages of HS lesions [156]. Anaerobic bacteria, mainly Gram-negative bacilli *Prevotella* and *Porphyromonas* spp. were also isolated in early and chronic HS lesions [153,154]. In addition, Enterobacteriaceae, particularly *Proteus mirabilis*, were commonly identified in cultures of superficial and deep lesions [153,157].

Thus, the most common species found on bacteriology analysis of superficial and deep lesions of HS patients are CoNS, *Staphylococcus aureus*, and anaerobic bacteria. These bacteria have been shown to be capable of biofilm formation, which might be responsible for resistance to antimicrobial therapy. Studies based on 16S and 18S ribosomal RNA (rRNA) next-generation sequencing have provided new insights into the role of the skin microbiome in the pathophysiology of HS, most likely a biofilm-driven disease [158]. It was demonstrated that there is a significantly different microbiome in HS patients, either lesional or non-lesional, compared to that in healthy controls [158]. In metagenomic studies, *Corynebacterium* spp., *Porphyromonas,* and *Peptoniphilus* ssp. were the predominant species identified from HS lesions, whereas *Porphyromonas* and *Peptoniphilus* ssp. were not detected in healthy control. In contrast, healthy skin showed a relatively higher abundance of *Propionibacterium* ssp. [159,160]. Some studies have shown that a mixed anaerobic microbiome was associated with clinical severity, and the abundance of anaerobes *Fusobacterium* and *Parvimonas* increases with higher classifications of Hurley staging [161,162]. These studies, while limited, indicate a proliferative bacterial phenotype in HS lesions. Ring et al. have specifically investigated the microbiome in the persistently inflamed sinus tracts of moderate and severe HS patients, finding that these samples were dominated by *Prevotella* ssp., *Porphyromonas* ssp., and other anaerobic species [163].

In conclusion, a pattern of cutaneous dysbiosis appears in HS, which highlights the presence of anaerobic bacteria and *Staphylococcus* spp., but the pathogenic role of the microbiome in HS is still unclear. The presence of microorganisms in HS has led to the condition being considered an infectious disorder. This is reinforced by the current guidelines for therapy focusing on the use of antibiotics [150,164].

## 4. Discussion

Hidradenitis suppurativa is a condition that involves several, concomitant pathways and, even though its pathogenesis remains unclear, more and more efforts are being made to elucidate the trigger factors of this debilitating disease. Various cytokines seem to be involved in the pathogenesis of HS, and the dysregulation of multiple inflammatory pathways, such as TNF, IL-1, IL-17/23, and anti-inflammatory cytokines, such as IL-10, has been observed.

Although none of the current treatments seem to adequately control this condition, immunomodulatory treatments targeting the Th17 pathway and the JAK/STAT pathways are now explored in HS. Anti-IL-17 drugs such as secukinumab, bimekizumab, or brodalumab and anti-IL-23 drugs such as risankizumab or guselkumab may represent an effective alternative in controlling this condition. Other biologic drugs directed to neutralize different axes have also been explored. The anti-IL-1 drug, bermekimab, it is currently under investigation, and may also become a treatment opportunity for patients with moderate-to-severe HS. Although the trials for the majority of this cytokine’s selective inhibitors are still at early phases (most of the clinical trials are in phase two or three), the pursuit of an effective treatment for the most severe cases of HS seems to have promising alternatives that are at present under investigation.

## Figures and Tables

**Table 1:** Completed interventional studies on target therapies.

NCT Clinical Trial	Intervention	Phase	Study Design	Enrollment
NCT03512275 [13]	Bermekimab 400 mg	Phase 2	•Allocation: Non-Randomized	42
			•Intervention Model: Single Group Assignment	
			•Masking: None (Open Label)	
NCT03960268 [14]	Brodalumab	Phase 1	•Allocation: N/A	10
			•Intervention Model: Single Group Assignment	
			•Masking: None (Open Label)	
NCT03607487 [15]	INCB054707	Phase 2	•Allocation: Randomized	36
	Placebo		•Intervention Model: Parallel Assignment	
			•Masking: Triple	
NCT03569371 [16]	INCB054707	Phase 2	•Allocation: N/A	10
			•Intervention Model: Single Group Assignment	
			•Masking: None (Open Label)	
NCT03248531 [17]	Bimekizumab	Phase 2	•Allocation: Randomized	90
	Adalimumab		•Intervention Model: Parallel Assignment	
	Placebo		•Masking: Quadruple	
NCT01516749 [18]	Anakinra	Phase 2	•Allocation: N/A •Intervention Model: Single Group Assignment •Masking: None (Open Label)	6
NCT02421172 [19]	CJM112	Phase 2	•Allocation: Randomized	66
	Placebo		•Intervention Model: Parallel Assignment	
			•Masking: Double (Participant, Investigator)	
NCT00795574 [20]	Infliximab	Phase 2	•Allocation: Randomized	38
	Placebo Comparator		•Intervention Model: Crossover Assignment	
			•Masking: Quadruple	
NCT00329823 [21]	Etanercept sc 50 mg per week for 12 weeks	Phase 2	•Allocation: Non-Randomized	10
			•Intervention Model: Single Group Assignment	
			•Masking: None (Open Label)	
NCT03628924 [22]	Guselkumab dose 1	Phase 2	•Allocation: Randomized	184
	Guselkumab dose 2		•Intervention Model: Parallel Assignment	
	Guselkumab dose 3		•Masking: Double (Participant, Investigator)	
NCT03001622 [23]	IFX-1	Phase 2	•Allocation: N/A	12
			•Intervention Model: Single Group Assignment	
			•Masking: None (Open Label)	
NCT03049267 [24]	Apremilast	Phase 2	•Allocation: Randomized	20
	Placebo Oral Tablet		•Intervention Model: Parallel Assignment	
			•Masking: Double (Participant, Investigator)	
NCT03099980 [25]	Secukinumab	Phase 1	•Allocation: N/A •Intervention Model: Single Group Assignment •Masking: None (Open Label)	20
NCT00107991 [26]	Etanercept	Phase 2	•Allocation: N/A	15
			•Intervention Model: Single Group Assignment	
			•Masking: None (Open Label)	
NCT02904902 [27]	Adalimumab	Phase 3	•Allocation: N/A	15
			•Intervention Model: Single Group Assignment	
			•Masking: None (Open Label)	
NCT02643654 [28]	MABp1	Phase 2	•Allocation: Randomized	20
	Placebo		•Intervention Model: Parallel Assignment	
			•Masking: Quadruple	
NCT02695212 [29]	Apremilast	Phase 2	•Allocation: N/A •Intervention Model: Single Group Assignment •Masking: None (Open Label)	20
NCT01704534 [30]	Ustekinumab	Phase 2	•Allocation: N/A	20
			•Intervention Model: Single Group Assignment	
			•Masking: None (Open Label)	
NCT03487276 [31]	IFX-1	Phase 2	•Allocation: Randomized	179
	Placebo		•Intervention Model: Parallel Assignment	
			•Masking: Quadruple	
NCT01558375 [32]	Anakinra	Phase 2	•Allocation: Randomized	20
	Water for injection		•Intervention Model: Parallel Assignment	
			•Masking: Quadruple	
NCT01635764 [33]	Adalimumab	Phase 3	•Allocation: N/A •Intervention Model: Single Group Assignment •Masking: None (Open Label)	508
NCT02808975 [34]	Adalimumab	Phase 4	•Allocation: Randomized	206
	Placebo		•Intervention Model: Parallel Assignment	
			•Masking: Quadruple	
NCT00918255 [35]	Adalimumab	Phase 2	•Allocation: Randomized	154
	Placebo		•Intervention Model: Parallel Assignment	
			•Masking: Quadruple	
NCT01468207 [36]	Adalimumab	Phase 3	•Allocation: Randomized	307
	placebo		•Intervention Model: Parallel Assignment	
			•Masking: Double (Participant, Investigator)	
NCT01468233 [37]	Adalimumab	Phase 3	•Allocation: Randomized	326
	placebo		•Intervention Model: Parallel Assignment	
			•Masking: Double (Participant, Investigator)	
NCT00827996 [38]	Adalimumab	Phase 2	•Allocation: N/A	10
			•Intervention Model: Single Group Assignment	
			•Masking: None (Open Label)	
NCT04018599 [39]	40 mg MSB11022	Phase 1	•Allocation: Randomized	216
			•Intervention Model: Parallel Assignment	
			•Masking: None (Open Label)	

IFX, infliximab.

**Table 2:** Recruiting interventional studies on target therapies.

NCT Clinical Trial	Intervention	Phase	Study Design	Enrollment
NCT03512275 [13]	CFZ533	Phase 2	•Allocation: Randomized	90
	LY006		•Intervention Model: Parallel Assignment	
	Placebo		•Masking: Quadruple	
NCT03926169 [40]	Risankizumab	Phase 2	•Allocation: Randomized	220
	Placebo		•Intervention Model: Parallel Assignment	
			•Masking: Quadruple	
NCT04430855 [41]	Upadacitinib	Phase 2	•Allocation: Randomized	60
	Placebo		•Intervention Model: Parallel Assignment	
			•Masking: Quadruple	
NCT04242498 [42]	Bimekizumab	Phase 3	•Allocation: Randomized	460
	Placebo		•Intervention Model: Parallel Assignment	
			•Masking: Quadruple	
NCT04179175 [43]	Secukinumab	Phase 3	•Allocation: Randomized	745
			•Intervention Model: Parallel Assignment	
			•Masking: Triple	
NCT03713632 [44]	Secukinumab Placebo	Phase 3	•Allocation: N/A •Intervention Model: Parallel Assignment •Masking: Triple	471
NCT04092452 [45]	PF-06650833, Placebo	Phase 2	•Allocation: Randomized	192
	PF-06700841		•Intervention Model: Parallel Assignment	
	PF-06826647		•Masking: Triple	
NCT04246372 [46]	Tofacitinib	Phase 2	•Allocation: N/A	46
			•Intervention Model: Single Group Assignment	
			•Masking: None (Open Label)	

**Table 3:** Recruiting interventional studies on target therapies.

Cytokines	Drugs	Quality of Evidence
anti-TNF-α	Adalimumab [33,34,35,36,37,38]	A
	Infliximab [20]	B
	Etanercept [26]	B
anti-IL-1	Anakinra [18]	B
	MEDI8968	Ongoing Trial
	Canakinumab	C
	Bermekimab [13]	B
anti-IL-12/23	Ustekinumab [30]	Ongoing Trial
anti-IL-23	Guselkumab [22]	Ongoing Trial
	Risankizumab [40]	Ongoing Trial
anti-IL-17	Secukinumab [43,44]	Ongoing Trial
	CJM112 [19]	Ongoing Trial
	Bimekizumab [42]	Ongoing Trial
	Brodalumab [14,16]	Ongoing Trial
anti-PDE-4	Apremilast [24]	B
anti-C5a	IFX-1 [31]	Ongoing Trial
anti-CD20	Rituximab	C
anti-CD40	Iscalimab [13]	Ongoing Trial
anti-JAK	Upadacitinib [41]	Ongoing Trial
	INCB054707 [15,16]	Ongoing Trial
	Tofacitinib [46]	Ongoing Trial

TNF, tumor necrosis factor; IL, interleukin; JAK, Janus kinase; PDE, phosphodiesterase.

## References

[1] Goldburg S.R., Strober B.E., Payette M.J. (2020). Hidradenitis suppurativa: Epidemiology, clinical presentation, and pathogenesis. J. Am. Acad. Dermatol..

[2] Del Duca E., Pavel A.B., Dubin C., Song T., Wallace E.B., Peng X., Estrada Y.D., Xu H., Maari C., Jack C. (2019). Major Differences in Expression of Inflammatory Pathways in Skin from Different Body Sites of Healthy Individuals. J. Investig. Dermatol..

[3] Chiricozzi A., Giovanardi G., Caro D.R.C., Iannone M., Garcovich S., Dini V., De Simone C., Franceschini C., Oranges T., Mingrone G. (2018). Alexithymia affects patients with hidradenitis suppurativa. Eur. J. Dermatol..

[4] Cusack C., Buckley C. (2006). Etanercept: Effective in the management of hidradenitis suppurativa. Br. J. Dermatol..

[5] Kraft J.N., Searles G.E. (2007). Hidradenitis suppurativa in 64 female patients: Retrospective study comparing oral antibiotics and antiandrogen therapy. J. Cutan. Med. Surg..

[6] Jemec G.B., Wendelboe P. (1998). Topical clindamycin versus systemic tetracycline in the treatment of hidradenitis suppurativa. J. Am. Acad. Dermatol..

[7] Strober B.E., Kim C., Siu K. (2007). Efalizumab for the treatment of refractory hidradenitis suppurativa. J. Am. Acad. Dermatol..

[8] Finley E.M., Ratz J.L. (1996). Treatment of hidradenitis suppurativa with carbon dioxide laser excision and second-intention healing. J. Am. Acad. Dermatol..

[9] Nistico S.P., Del Duca E., Farnetani F., Guida S., Pellacani G., Rajabi-Estarabadi A., Nouri K. (2018). Removal of unwanted hair: Efficacy, tolerability, and safety of long-pulsed 755-nm alexandrite laser equipped with a sapphire handpiece. Lasers Med. Sci..

[10] van der Zee H.H., Prens E.P., Boer J. (2010). Deroofing: A tissue-saving surgical technique for the treatment of mild to moderate hidradenitis suppurativa lesions. J. Am. Acad. Dermatol..

[11] Sabat R., Jemec G.B.E., Matusiak L., Kimball A.B., Prens E., Wolk K. (2020). Hidradenitis suppurativa. Nat. Rev. Dis. Primers.

[12] Kurokawa I., Nishijima S., Kusumoto K., Senzaki H., Shikata N., Tsubura A. (2002). Immunohistochemical study of cytokeratins in hidradenitis suppurativa (acne inversa). J. Int. Med. Res..

[13] A Study of Bermekimab in Patients with Hidradenitis Suppurativa. https://ClinicalTrials.gov/show/NCT03512275.

[14] Biomarkers in Hidradenitis Suppurativa Participants Receiving Brodalumab. https://ClinicalTrials.gov/show/NCT03960268.

[15] A Placebo-Controlled Study of the Safety of INCB054707 in Participants with Hidradenitis Suppurativa. https://ClinicalTrials.gov/show/NCT03607487.

[16] A Study of the Safety of INCB054707 in Participants with Hidradenitis Suppurativa. https://ClinicalTrials.gov/show/NCT03569371.

[17] A Study to Test the Efficacy, Safety and Pharmacokinetics of Bimekizumab in Subjects with Moderate to Severe Hidradenitis Suppurativa. https://ClinicalTrials.gov/show/NCT03248531.

[18] Anakinra as a Treatment for Hydradenitis Suppurativa. https://ClinicalTrials.gov/show/NCT01516749.

[19] Efficacy, Safety, and Pharmacokinetics Study of CJM112 in Hidradenitis Suppurativa Patients. https://ClinicalTrials.gov/show/NCT02421172.

[20] Study to Assess the Safety and Efficacy of Infliximab to Treat Hidradenitis Suppurtativa. https://ClinicalTrials.gov/show/NCT00795574.

[21] Etanercept in Hidradenitis Suppurativa. https://ClinicalTrials.gov/show/NCT00329823.

[22] A Study to Evaluate the Efficacy, Safety, and Tolerability of Guselkumab for the Treatment of Participants with Moderate to Severe Hidradenitis Suppurativa (HS). https://ClinicalTrials.gov/show/NCT03628924.

[23] Studying Complement Inhibition in Patients with Moderate to Severe Hidradenitis Suppurativa. https://ClinicalTrials.gov/show/NCT03001622.

[24] Short-Term Safety, Efficacy and Mode of Action of Apremilast in Moderate Suppurative Hidradenitis. https://ClinicalTrials.gov/show/NCT03049267.

[25] Exploratory Trial Evaluating Cosentyx (Secukinumab) for Patients with Moderate-To-Severe Hidradenitis Suppurativa. https://ClinicalTrials.gov/show/NCT03099980.

[26] Etanercept for Treatment of Hidradenitis. https://ClinicalTrials.gov/show/NCT00107991.

[27] Open-Label Study of Adalimumab in Japanese Subjects with Hidradenitis Suppurativa. https://ClinicalTrials.gov/show/NCT02904902.

[28] MABP1 in Hidradenitis Suppurativa Refractory to Adalimumab. https://ClinicalTrials.gov/show/NCT02643654.

[29] Single Center Study of Apremilast for the Treatment of Hidradenitis Suppurativa. https://ClinicalTrials.gov/show/NCT02695212.

[30] A Proof of Concept Study to Evaluate the Effectiveness of Ustekinumab in Hidradenitis Suppurativa. https://ClinicalTrials.gov/show/NCT01704534.

[31] Efficacy and Safety Study of IFX-1 in Patients with Moderate to Severe Hidradenitis Suppurativa (HS). https://ClinicalTrials.gov/show/NCT03487276.

[32] Anakinra in Hidradenitis Suppurativa. https://ClinicalTrials.gov/show/NCT01558375.

[33] Open-Label Study of the Safety and Efficacy of Adalimumab in the Treatment of Hidradenitis Suppurativa. https://ClinicalTrials.gov/show/NCT01635764.

[34] Safety and Efficacy of Adalimumab (Humira) for Hidradenitis Suppurativa (HS) Peri-Surgically. https://ClinicalTrials.gov/show/NCT02808975.

[35] Study of Adalimumab in Subjects with Moderate to Severe Chronic Hidradenitis Suppurativa. https://ClinicalTrials.gov/show/NCT00918255.

[36] Efficacy and Safety Study of Adalimumab in Treatment of Hidradenitis Suppurativa. https://ClinicalTrials.gov/show/NCT01468207.

[37] Efficacy and Safety Study of Adalimumab in the Treatment of Hidradenitis Suppurativa. https://ClinicalTrials.gov/show/NCT01468233.

[38] To Assess the Efficacy and Safety of Adalimumab in Subjects with Moderate to Severe Hidradenitis Suppurativa. https://ClinicalTrials.gov/show/NCT00827996.

[39] Comparison of PK and Tolerability of MSB11022 Administered by AI or PFS. https://ClinicalTrials.gov/show/NCT04018599.

[40] A Global Study Comparing Risankizumab to Placebo in Adult Participants with Moderate to Severe Hidradenitis Suppurativa. https://ClinicalTrials.gov/show/NCT03926169.

[41] A Study of Oral Upadacitinib Tablet Compared to Placebo in Adult Participants with Moderate to Severe Hidradenitis Suppurativa to Assess Change in Disease Symptoms. https://ClinicalTrials.gov/show/NCT04430855.

[42] A Study to Test the Efficacy and Safety of Bimekizumab in Study Participants with Moderate to Severe Hidradenitis Suppurativa. https://ClinicalTrials.gov/show/NCT04242498.

[43] Extension Study to Assess Effects of Non-Interrupted Versus Interrupted and Long Term Treatment of Two Dose Regimes of Secukinumab in Subjects with Hidradenitis Suppurativa. https://ClinicalTrials.gov/show/NCT04179175.

[44] Study of Efficacy and Safety of Two Secukinumab Dose Regimens in Subjects with Moderate to Severe Hidradenitis Suppurativa (HS). https://ClinicalTrials.gov/show/NCT03713632.

[45] A Study to Evaluate the Safety and Efficacy of PF-06650833, PF-06700841, and PF 06826647 in Adults with Hidradenitis Suppurativa. https://ClinicalTrials.gov/show/NCT04092452.

[46] Tofacitinib for Immune Skin Conditions in Down Syndrome. https://ClinicalTrials.gov/show/NCT04246372.

[47] Wolk K., Join-Lambert O., Sabat R. (2020). Aetiology and pathogenesis of hidradenitis suppurativa. Br. J. Dermatol..

[48] Frew J.W., Hawkes J.E., Krueger J.G. (2019). Topical, systemic and biologic therapies in hidradenitis suppurativa: Pathogenic insights by examining therapeutic mechanisms. Ther. Adv. Chronic. Dis..

[49] Vossen A., van der Zee H.H., Prens E.P. (2018). Hidradenitis Suppurativa: A Systematic Review Integrating Inflammatory Pathways Into a Cohesive Pathogenic Model. Front. Immunol..

[50] van der Zee H.H., de Ruiter L., Boer J., van den Broecke D.G., den Hollander J.C., Laman J.D., Prens E.P. (2012). Alterations in leucocyte subsets and histomorphology in normal-appearing perilesional skin and early and chronic hidradenitis suppurativa lesions. Br. J. Dermatol..

[51] Tsaousi A., Witte E., Witte K., Rowert-Huber H.J., Volk H.D., Sterry W., Wolk K., Schneider-Burrus S., Sabat R. (2016). MMP8 Is Increased in Lesions and Blood of Acne Inversa Patients: A Potential Link to Skin Destruction and Metabolic Alterations. Mediat. Inflamm..

[52] Mozeika E., Pilmane M., Nurnberg B.M., Jemec G.B. (2013). Tumour necrosis factor-alpha and matrix metalloproteinase-2 are expressed strongly in hidradenitis suppurativa. Acta Derm. Venereol..

[53] Hoffman L.K., Ghias M.H., Lowes M.A. (2017). Pathophysiology of hidradenitis suppurativa. Semin. Cutan. Med. Surg..

[54] Ring H.C., Bay L., Nilsson M., Kallenbach K., Miller I.M., Saunte D.M., Bjarnsholt T., Tolker-Nielsen T., Jemec G.B. (2017). Bacterial biofilm in chronic lesions of hidradenitis suppurativa. Br. J. Dermatol..

[55] Giamarellos-Bourboulis E.J., Antonopoulou A., Petropoulou C., Mouktaroudi M., Spyridaki E., Baziaka F., Pelekanou A., Giamarellou H., Stavrianeas N.G. (2007). Altered innate and adaptive immune responses in patients with hidradenitis suppurativa. Br. J. Dermatol..

[56] van der Zee H.H., de Ruiter L., van den Broecke D.G., Dik W.A., Laman J.D., Prens E.P. (2011). Elevated levels of tumour necrosis factor (TNF)-alpha, interleukin (IL)-1beta and IL-10 in hidradenitis suppurativa skin: A rationale for targeting TNF-alpha and IL-1beta. Br. J. Dermatol..

[57] von Laffert M., Helmbold P., Wohlrab J., Fiedler E., Stadie V., Marsch W.C. (2010). Hidradenitis suppurativa (acne inversa): Early inflammatory events at terminal follicles and at interfollicular epidermis. Exp. Dermatol..

[58] Seyed Jafari S.M., Hunger R.E., Schlapbach C. (2020). Hidradenitis Suppurativa: Current Understanding of Pathogenic Mechanisms and Suggestion for Treatment Algorithm. Front. Med..

[59] Kelly G., Sweeney C.M., Tobin A.M., Kirby B. (2014). Hidradenitis suppurativa: The role of immune dysregulation. Int. J. Dermatol..

[60] Kelly G., Hughes R., McGarry T., van den Born M., Adamzik K., Fitzgerald R., Lawlor C., Tobin A.M., Sweeney C.M., Kirby B. (2015). Dysregulated cytokine expression in lesional and nonlesional skin in hidradenitis suppurativa. Br. J. Dermatol..

[61] van der Zee H.H., Laman J.D., Boer J., Prens E.P. (2012). Hidradenitis suppurativa: Viewpoint on clinical phenotyping, pathogenesis and novel treatments. Exp. Dermatol..

[62] Franza L., Carusi V., Altamura S., Caraffa A., Gallenga C.E., Kritas S.K., Ronconi G., Conti P., Pandolfi F. (2019). Interrelationship between inflammatory cytokines (IL-1, IL-6, IL-33, IL-37) and acquired immunity. J. Biol. Regul. Homeost. Agents.

[63] Caraffa A., Gallenga C.E., Kritas S.K., Ronconi G., Di Emidio P., Conti P. (2019). CAR-T cell therapy causes inflammation by IL-1 which activates inflammatory cytokine mast cells: Anti-inflammatory role of IL-37. J. Biol. Regul. Homeost. Agents.

[64] Dinarello C.A. (2018). Overview of the IL-1 family in innate inflammation and acquired immunity. Immunol. Rev..

[65] Conti P., Caraffa A., Gallenga C.E., Ross R., Kritas S.K., Frydas I., Younes A., Di Emidio P., Ronconi G., Toniato E. (2020). IL-1 induces throboxane-A2 (TxA2) in COVID-19 causing inflammation and micro-thrombi: Inhibitory effect of the IL-1 receptor antagonist (IL-1Ra). J. Biol. Regul. Homeost. Agents.

[66] Conti P., Gallenga C.E., Tete G., Caraffa A., Ronconi G., Younes A., Toniato E., Ross R., Kritas S.K. (2020). How to reduce the likelihood of coronavirus-19 (CoV-19 or SARS-CoV-2) infection and lung inflammation mediated by IL-1. J. Biol. Regul. Homeost. Agents.

[67] Witte-Handel E., Wolk K., Tsaousi A., Irmer M.L., Mossner R., Shomroni O., Lingner T., Witte K., Kunkel D., Salinas G. (2019). The IL-1 Pathway Is Hyperactive in Hidradenitis Suppurativa and Contributes to Skin Infiltration and Destruction. J. Investig. Dermatol..

[68] Hessam S., Sand M., Gambichler T., Bechara F.G. (2015). Correlation of inflammatory serum markers with disease severity in patients with hidradenitis suppurativa (HS). J. Am. Acad. Dermatol..

[69] Jorch S.K., Kubes P. (2017). An emerging role for neutrophil extracellular traps in noninfectious disease. Nat. Med..

[70] Ardon C.B., Wang C., Prens E.P., van Straalen K.R. (2020). Non-invasive assessment of cytokine and antimicrobial peptide levels in Hidradenitis Suppurativa using transdermal analysis patches. Br. J. Dermatol..

[71] Queen D., Ediriweera C., Liu L. (2019). Function and Regulation of IL-36 Signaling in Inflammatory Diseases and Cancer Development. Front. Cell Dev. Biol..

[72] Hessam S., Sand M., Gambichler T., Skrygan M., Ruddel I., Bechara F.G. (2018). Interleukin-36 in hidradenitis suppurativa: Evidence for a distinctive proinflammatory role and a key factor in the development of an inflammatory loop. Br. J. Dermatol..

[73] Di Caprio R., Balato A., Caiazzo G., Lembo S., Raimondo A., Fabbrocini G., Monfrecola G. (2017). IL-36 cytokines are increased in acne and hidradenitis suppurativa. Arch. Dermatol. Res..

[74] Teng X., Hu Z., Wei X., Wang Z., Guan T., Liu N., Liu X., Ye N., Deng G., Luo C. (2014). IL-37 ameliorates the inflammatory process in psoriasis by suppressing proinflammatory cytokine production. J. Immunol..

[75] van de Veerdonk F.L., Stoeckman A.K., Wu G., Boeckermann A.N., Azam T., Netea M.G., Joosten L.A., van der Meer J.W., Hao R., Kalabokis V. (2012). IL-38 binds to the IL-36 receptor and has biological effects on immune cells similar to IL-36 receptor antagonist. Proc. Natl. Acad. Sci. USA.

[76] Frew J.W., Hawkes J.E., Krueger J.G. (2018). A systematic review and critical evaluation of inflammatory cytokine associations in hidradenitis suppurativa. F1000Research.

[77] Shah A., Alhusayen R., Amini-Nik S. (2017). The critical role of macrophages in the pathogenesis of hidradenitis suppurativa. Inflamm. Res..

[78] Savage K.T., Flood K.S., Porter M.L., Kimball A.B. (2019). TNF-alpha inhibitors in the treatment of hidradenitis suppurativa. Ther. Adv. Chronic. Dis..

[79] Kyriakou A., Trigoni A., Galanis N., Sotiriadis D., Patsatsi A. (2018). Efficacy of adalimumab in moderate to severe hidradenitis suppurativa: Real life data. Dermatol. Rep..

[80] Moran B., Sweeney C.M., Hughes R., Malara A., Kirthi S., Tobin A.M., Kirby B., Fletcher J.M. (2017). Hidradenitis Suppurativa Is Characterized by Dysregulation of the Th17:Treg Cell Axis, Which Is Corrected by Anti-TNF Therapy. J. Investig. Dermatol..

[81] Vossen A., van der Zee H.H., Tsoi L.C., Xing X., Devalaraja M., Gudjonsson J.E., Prens E.P. (2019). Novel cytokine and chemokine markers of hidradenitis suppurativa reflect chronic inflammation and itch. Allergy.

[82] Schroder K., Hertzog P.J., Ravasi T., Hume D.A. (2004). Interferon-gamma: An overview of signals, mechanisms and functions. J. Leukoc. Biol..

[83] Banerjee A., McNish S., Shanmugam V.K. (2017). Interferon-gamma (IFN-gamma) is Elevated in Wound Exudate from Hidradenitis Suppurativa. Immunol. Investig..

[84] Wolk K., Warszawska K., Hoeflich C., Witte E., Schneider-Burrus S., Witte K., Kunz S., Buss A., Roewert H.J., Krause M. (2011). Deficiency of IL-22 contributes to a chronic inflammatory disease: Pathogenetic mechanisms in acne inversa. J. Immunol..

[85] Frew J.W., Navrazhina K., Grand D., Sullivan-Whalen M., Gilleaudeau P., Garcet S., Ungar J., Krueger J.G. (2020). The effect of subcutaneous brodalumab on clinical disease activity in hidradenitis suppurativa: An open-label cohort study. J. Am. Acad. Dermatol..

[86] Schlapbach C., Hanni T., Yawalkar N., Hunger R.E. (2011). Expression of the IL-23/Th17 pathway in lesions of hidradenitis suppurativa. J. Am. Acad. Dermatol..

[87] Bunte K., Beikler T. (2019). Th17 Cells and the IL-23/IL-17 Axis in the Pathogenesis of Periodontitis and Immune-Mediated Inflammatory Diseases. Int. J. Mol. Sci..

[88] Monin L., Gaffen S.L. (2018). Interleukin 17 Family Cytokines: Signaling Mechanisms, Biological Activities, and Therapeutic Implications. Cold Spring Harb. Perspect. Biol..

[89] Hotz C., Boniotto M., Guguin A., Surenaud M., Jean-Louis F., Tisserand P., Ortonne N., Hersant B., Bosc R., Poli F. (2016). Intrinsic Defect in Keratinocyte Function Leads to Inflammation in Hidradenitis Suppurativa. J. Investig. Dermatol..

[90] Lima A.L., Karl I., Giner T., Poppe H., Schmidt M., Presser D., Goebeler M., Bauer B. (2016). Keratinocytes and neutrophils are important sources of proinflammatory molecules in hidradenitis suppurativa. Br. J. Dermatol..

[91] Matusiak L., Szczech J., Bieniek A., Nowicka-Suszko D., Szepietowski J.C. (2017). Increased interleukin (IL)-17 serum levels in patients with hidradenitis suppurativa: Implications for treatment with anti-IL-17 agents. J. Am. Acad. Dermatol..

[92] Thomi R., Schlapbach C., Yawalkar N., Simon D., Yerly D., Hunger R.E. (2018). Elevated levels of the antimicrobial peptide LL-37 in hidradenitis suppurativa are associated with a Th1/Th17 immune response. Exp. Dermatol..

[93] Yao Y., Thomsen S.F. (2017). The role of interleukin-17 in the pathogenesis of hidradenitis suppurativa. Dermatol. Online J..

[94] Zheng Y., Danilenko D.M., Valdez P., Kasman I., Eastham-Anderson J., Wu J., Ouyang W. (2007). Interleukin-22, a T(H)17 cytokine, mediates IL-23-induced dermal inflammation and acanthosis. Nature.

[95] Lyakh L., Trinchieri G., Provezza L., Carra G., Gerosa F. (2008). Regulation of interleukin-12/interleukin-23 production and the T-helper 17 response in humans. Immunol. Rev..

[96] Tanaka T., Kishimoto T. (2012). Targeting interleukin-6: All the way to treat autoimmune and inflammatory diseases. Int. J. Biol. Sci..

[97] Bechara F.G., Sand M., Skrygan M., Kreuter A., Altmeyer P., Gambichler T. (2012). Acne inversa: Evaluating antimicrobial peptides and proteins. Ann. Dermatol..

[98] Dreno B., Khammari A., Brocard A., Moyse D., Blouin E., Guillet G., Leonard F., Knol A.C. (2012). Hidradenitis suppurativa: The role of deficient cutaneous innate immunity. Arch. Dermatol..

[99] Xu H., Xiao X., He Y., Zhang X., Li C., Mao Q., Wu X., Wang B. (2017). Increased serum interleukin-6 levels in patients with hidradenitis suppurativa. Postepy Dermatol. Alergol..

[100] Chaudhry A., Samstein R.M., Treuting P., Liang Y., Pils M.C., Heinrich J.M., Jack R.S., Wunderlich F.T., Bruning J.C., Muller W. (2011). Interleukin-10 signaling in regulatory T cells is required for suppression of Th17 cell-mediated inflammation. Immunity.

[101] Iyer S.S., Cheng G. (2012). Role of interleukin 10 transcriptional regulation in inflammation and autoimmune disease. Crit. Rev. Immunol..

[102] Fukaya T., Fukui T., Uto T., Takagi H., Nasu J., Miyanaga N., Arimura K., Nakamura T., Koseki H., Choijookhuu N. (2018). Pivotal Role of IL-22 Binding Protein in the Epithelial Autoregulation of Interleukin-22 Signaling in the Control of Skin Inflammation. Front. Immunol..

[103] Sabat R., Ouyang W., Wolk K. (2014). Therapeutic opportunities of the IL-22-IL-22R1 system. Nat. Rev. Drug Discov..

[104] Jones D., Banerjee A., Berger P.Z., Gross A., McNish S., Amdur R., Shanmugam V.K. (2018). Inherent differences in keratinocyte function in hidradenitis suppurativa: Evidence for the role of IL-22 in disease pathogenesis. Immunol. Investig..

[105] Jimenez-Gallo D., de la Varga-Martinez R., Ossorio-Garcia L., Albarran-Planelles C., Rodriguez C., Linares-Barrios M. (2017). The Clinical Significance of Increased Serum Proinflammatory Cytokines, C-Reactive Protein, and Erythrocyte Sedimentation Rate in Patients with Hidradenitis Suppurativa. Mediat. Inflamm..

[106] Wolk K., Wenzel J., Tsaousi A., Witte-Handel E., Babel N., Zelenak C., Volk H.D., Sterry W., Schneider-Burrus S., Sabat R. (2017). Lipocalin-2 is expressed by activated granulocytes and keratinocytes in affected skin and reflects disease activity in acne inversa/hidradenitis suppurativa. Br. J. Dermatol..

[107] Kimball A.B., Sobell J.M., Zouboulis C.C., Gu Y., Williams D.A., Sundaram M., Teixeira H.D., Jemec G.B. (2016). HiSCR (Hidradenitis Suppurativa Clinical Response): A novel clinical endpoint to evaluate therapeutic outcomes in patients with hidradenitis suppurativa from the placebo-controlled portion of a phase 2 adalimumab study. J. Eur. Acad. Dermatol. Venereol..

[108] Robinson J.K., Dellavalle R.P., Bigby M., Callen J.P. (2008). Systematic reviews: Grading recommendations and evidence quality. Arch. Dermatol..

[109] Gupta A.K., Studholme C. (2016). Adalimumab (Humira) for the Treatment of Hidradenitis Suppurativa. Skin Therapy Lett..

[110] Kimball A.B., Okun M.M., Williams D.A., Gottlieb A.B., Papp K.A., Zouboulis C.C., Armstrong A.W., Kerdel F., Gold M.H., Forman S.B. (2016). Two Phase 3 Trials of Adalimumab for Hidradenitis Suppurativa. N. Engl. J. Med..

[111] Menter A., Tyring S.K., Gordon K., Kimball A.B., Leonardi C.L., Langley R.G., Strober B.E., Kaul M., Gu Y., Okun M. (2008). Adalimumab therapy for moderate to severe psoriasis: A randomized, controlled phase III trial. J. Am. Acad. Dermatol..

[112] Knight D.M., Trinh H., Le J., Siegel S., Shealy D., McDonough M., Scallon B., Moore M.A., Vilcek J., Daddona P. (1993). Construction and initial characterization of a mouse-human chimeric anti-TNF antibody. Mol. Immunol..

[113] Ghias M.H., Johnston A.D., Kutner A.J., Micheletti R.G., Hosgood H.D., Cohen S.R. (2020). High-dose, high-frequency infliximab: A novel treatment paradigm for hidradenitis suppurativa. J. Am. Acad. Dermatol..

[114] Grant A., Gonzalez T., Montgomery M.O., Cardenas V., Kerdel F.A. (2010). Infliximab therapy for patients with moderate to severe hidradenitis suppurativa: A randomized, double-blind, placebo-controlled crossover trial. J. Am. Acad. Dermatol..

[115] Orenstein L.A.V., Nguyen T.V., Damiani G., Sayed C., Jemec G.B.E., Hamzavi I. (2020). Medical and Surgical Management of Hidradenitis Suppurativa: A Review of International Treatment Guidelines and Implementation in General Dermatology Practice. Dermatology.

[116] Moreland L.W., Baumgartner S.W., Schiff M.H., Tindall E.A., Fleischmann R.M., Weaver A.L., Ettlinger R.E., Cohen S., Koopman W.J., Mohler K. (1997). Treatment of rheumatoid arthritis with a recombinant human tumor necrosis factor receptor (p75)-Fc fusion protein. N. Engl. J. Med..

[117] Pelekanou A., Kanni T., Savva A., Mouktaroudi M., Raftogiannis M., Kotsaki A., Giamarellos-Bourboulis E.J. (2010). Long-term efficacy of etanercept in hidradenitis suppurativa: Results from an open-label phase II prospective trial. Exp. Dermatol..

[118] Lee R.A., Dommasch E., Treat J., Sciacca-Kirby J., Chachkin S., Williams J., Shin D.B., Leyden J.J., Vittorio C., Gelfand J.M. (2009). A prospective clinical trial of open-label etanercept for the treatment of hidradenitis suppurativa. J. Am. Acad. Dermatol..

[119] Tzanetakou V., Kanni T., Giatrakou S., Katoulis A., Papadavid E., Netea M.G., Dinarello C.A., van der Meer J.W.M., Rigopoulos D., Giamarellos-Bourboulis E.J. (2016). Safety and Efficacy of Anakinra in Severe Hidradenitis Suppurativa: A Randomized Clinical Trial. JAMA Dermatol..

[120] Andre R., Marescassier H., Gabay C., Pittet B., Laffitte E. (2019). Long-term therapy with anakinra in hidradenitis suppurativa in three patients. Int. J. Dermatol..

[121] Leslie K.S., Tripathi S.V., Nguyen T.V., Pauli M., Rosenblum M.D. (2014). An open-label study of anakinra for the treatment of moderate to severe hidradenitis suppurativa. J. Am. Acad. Dermatol..

[122] Russo V., Alikhan A. (2016). Failure of Anakinra in a Case of Severe Hidradenitis Suppurativa. J. Drugs Dermatol..

[123] Gottlieb A., Natsis N.E., Kerdel F., Forman S., Gonzalez E., Jimenez G., Hernandez L., Kaffenberger J., Guido G., Lucas K. (2020). A Phase II Open-Label Study of Bermekimab in Patients with Hidradenitis Suppurativa Shows Resolution of Inflammatory Lesions and Pain. J. Investig. Dermatol..

[124] Calverley P.M.A., Sethi S., Dawson M., Ward C.K., Finch D.K., Penney M., Newbold P., van der Merwe R. (2017). A randomised, placebo-controlled trial of anti-interleukin-1 receptor 1 monoclonal antibody MEDI8968 in chronic obstructive pulmonary disease. Respir. Res..

[125] Houriet C., Seyed Jafari S.M., Thomi R., Schlapbach C., Borradori L., Yawalkar N., Hunger R.E. (2017). Canakinumab for Severe Hidradenitis Suppurativa: Preliminary Experience in 2 Cases. JAMA Dermatol..

[126] Canakinumab (2006). Drugs and Lactation Database (LactMed).

[127] Lim S.Y.D., Oon H.H. (2019). Systematic review of immunomodulatory therapies for hidradenitis suppurativa. Biologics.

[128] Sun N.Z., Ro T., Jolly P., Sayed C.J. (2017). Non-response to Interleukin-1 Antagonist Canakinumab in Two Patients with Refractory Pyoderma Gangrenosum and Hidradenitis Suppurativa. J. Clin. Aesthet. Dermatol..

[129] Schuch A., Fischer T., Boehner A., Biedermann T., Volz T. (2018). Successful Treatment of Severe Recalcitrant Hidradenitis Suppurativa with the Interleukin-17A Antibody Secukinumab. Acta Derm. Venereol..

[130] Thorlacius L., Theut Riis P., Jemec G.B.E. (2018). Severe hidradenitis suppurativa responding to treatment with secukinumab: A case report. Br. J. Dermatol..

[131] Foulkes A.C., Warren R.B. (2019). Brodalumab in psoriasis: Evidence to date and clinical potential. Drugs Context.

[132] Zouboulis C.C., Tzellos T., Kyrgidis A., Jemec G.B.E., Bechara F.G., Giamarellos-Bourboulis E.J., Ingram J.R., Kanni T., Karagiannidis I., Martorell A. (2017). Development and validation of the International Hidradenitis Suppurativa Severity Score System (IHS4), a novel dynamic scoring system to assess HS severity. Br. J. Dermatol..

[133] Bai F., Li G.G., Liu Q., Niu X., Li R., Ma H. (2019). Short-Term Efficacy and Safety of IL-17, IL-12/23, and IL-23 Inhibitors Brodalumab, Secukinumab, Ixekizumab, Ustekinumab, Guselkumab, Tildrakizumab, and Risankizumab for the Treatment of Moderate to Severe Plaque Psoriasis: A Systematic Review and Network Meta-Analysis of Randomized Controlled Trials. J. Immunol. Res..

[134] Kovacs M., Podda M. (2019). Guselkumab in the treatment of severe hidradenitis suppurativa. J. Eur. Acad. Dermatol. Venereol..

[135] Takeda K., Kikuchi K., Kanazawa Y., Yamasaki K., Aiba S. (2019). Ustekinumab treatment for hidradenitis suppurativa. J. Dermatol..

[136] Blok J.L., Li K., Brodmerkel C., Horvatovich P., Jonkman M.F., Horvath B. (2016). Ustekinumab in hidradenitis suppurativa: Clinical results and a search for potential biomarkers in serum. Br. J. Dermatol..

[137] Montero-Vilchez T., Pozo-Roman T., Sanchez-Velicia L., Vega-Gutierrez J., Arias-Santiago S., Molina-Leyva A. (2020). Ustekinumab in the treatment of patients with hidradenitis suppurativa: Multicenter case series and systematic review. J. Dermatol. Treat..

[138] Valenzuela-Ubina S., Jimenez-Gallo D., Villegas-Romero I., Rodriguez-Mateos M.E., Linares-Barrios M. (2020). Effectiveness of ustekinumab for moderate-to-severe hidradenitis suppurativa: A case series. J. Dermatol. Treat..

[139] Serra Lopez-Matencio J.M., Morell Baladron A., Castaneda S. (2019). JAK-STAT inhibitors for the treatment of immunomediated diseases. Med. Clin..

[140] Savage K.T., Santillan M.R., Flood K.S., Charrow A., Porter M.L., Kimball A.B. (2020). Tofacitinib shows benefit in conjunction with other therapies in recalcitrant hidradenitis suppurativa patients. JAAD Case Rep..

[141] Genovese M.C., Smolen J.S., Weinblatt M.E., Burmester G.R., Meerwein S., Camp H.S., Wang L., Othman A.A., Khan N., Pangan A.L. (2016). Efficacy and Safety of ABT-494, a Selective JAK-1 Inhibitor, in a Phase IIb Study in Patients With Rheumatoid Arthritis and an Inadequate Response to Methotrexate. Arthritis Rheumatol..

[142] Bianchi L., Del Duca E., Romanelli M., Saraceno R., Chimenti S., Chiricozzi A. (2016). Pharmacodynamic assessment of apremilast for the treatment of moderate-to-severe plaque psoriasis. Expert Opin. Drug Metab. Toxicol..

[143] Dattola A., Del Duca E., Saraceno R., Gramiccia T., Bianchi L. (2017). Safety evaluation of apremilast for the treatment of psoriasis. Expert Opin. Drug Saf..

[144] Maloney N.J., Zhao J., Tegtmeyer K., Lee E.Y., Cheng K. (2020). Off-label studies on apremilast in dermatology: A review. J. Dermatol. Treat..

[145] Vossen A., van Doorn M.B.A., van der Zee H.H., Prens E.P. (2019). Apremilast for moderate hidradenitis suppurativa: Results of a randomized controlled trial. J. Am. Acad. Dermatol..

[146] Giamarellos-Bourboulis E.J., Argyropoulou M., Kanni T., Spyridopoulos T., Otto I., Zenker O., Guo R., Riedemann N.C. (2020). Clinical efficacy of complement C5a inhibition by IFX-1 in hidradenitis suppurativa: An open-label single-arm trial in patients not eligible for adalimumab. Br. J. Dermatol..

[147] Espie P., He Y., Koo P., Sickert D., Dupuy C., Chokote E., Schuler R., Mergentaler H., Ristov J., Milojevic J. (2020). First-in-human clinical trial to assess pharmacokinetics, pharmacodynamics, safety, and tolerability of iscalimab, an anti-CD40 monoclonal antibody. Am. J. Transpl..

[148] Kahaly G.J., Stan M.N., Frommer L., Gergely P., Colin L., Amer A., Schuhmann I., Espie P., Rush J.S., Basson C. (2020). A Novel Anti-CD40 Monoclonal Antibody, Iscalimab, for Control of Graves Hyperthyroidism-A Proof-of-Concept Trial. J. Clin. Endocrinol. Metab..

[149] Mavragani C.P., Moutsopoulos H.M. (2020). Sjogren’s syndrome: Old and new therapeutic targets. J. Autoimmun..

[150] Marasca C., Tranchini P., Marino V., Annunziata M.C., Napolitano M., Fattore D., Fabbrocini G. (2020). The pharmacology of antibiotic therapy in hidradenitis suppurativa. Expert Rev. Clin. Pharmacol..

[151] Hunger R.E., Surovy A.M., Hassan A.S., Braathen L.R., Yawalkar N. (2008). Toll-like receptor 2 is highly expressed in lesions of acne inversa and colocalizes with C-type lectin receptor. Br. J. Dermatol..

[152] Hessam S., Sand M., Georgas D., Anders A., Bechara F.G. (2016). Microbial Profile and Antimicrobial Susceptibility of Bacteria Found in Inflammatory Hidradenitis Suppurativa Lesions. Skin Pharmacol. Physiol..

[153] Bettoli V., Manfredini M., Massoli L., Carillo C., Barozzi A., Amendolagine G., Ruina G., Musmeci D., Libanore M., Curtolo A. (2019). Rates of antibiotic resistance/sensitivity in bacterial cultures of hidradenitis suppurativa patients. J. Eur. Acad. Dermatol. Venereol..

[154] Benzecry V., Grancini A., Guanziroli E., Nazzaro G., Barbareschi M., Marzano A.V., Muratori S., Veraldi S. (2018). Hidradenitis suppurativa/acne inversa: A prospective bacteriological study of 46 patients and review of the literature. G. Ital. Dermatol. Venereol..

[155] Sartorius K., Killasli H., Oprica C., Sullivan A., Lapins J. (2012). Bacteriology of hidradenitis suppurativa exacerbations and deep tissue cultures obtained during carbon dioxide laser treatment. Br. J. Dermatol..

[156] Ring H.C., Riis Mikkelsen P., Miller I.M., Jenssen H., Fuursted K., Saunte D.M., Jemec G.B. (2015). The bacteriology of hidradenitis suppurativa: A systematic review. Exp. Dermatol..

[157] Nikolakis G., Liakou A.I., Bonovas S., Seltmann H., Bonitsis N., Join-Lambert O., Wild T., Karagiannidis I., Zolke-Fischer S., Langner K. (2017). Bacterial Colonization in Hidradenitis Suppurativa/Acne Inversa: A Cross-sectional Study of 50 Patients and Review of the Literature. Acta Derm. Venereol..

[158] Ring H.C., Thorsen J., Jorgensen A.H., Bay L., Bjarnsholt T., Fuursted K., Thomsen S.F., Jemec G.B. (2020). Predictive Metagenomic Analysis Reveals a Role of Cutaneous Dysbiosis in the Development of Hidradenitis Suppurativa. J. Investig. Dermatol..

[159] Schneider A.M., Cook L.C., Zhan X., Banerjee K., Cong Z., Imamura-Kawasawa Y., Gettle S.L., Longenecker A.L., Kirby J.S., Nelson A.M. (2020). Loss of Skin Microbial Diversity and Alteration of Bacterial Metabolic Function in Hidradenitis Suppurativa. J. Investig. Dermatol..

[160] Ring H.C., Thorsen J., Saunte D.M., Lilje B., Bay L., Riis P.T., Larsen N., Andersen L.O., Nielsen H.V., Miller I.M. (2017). The Follicular Skin Microbiome in Patients With Hidradenitis Suppurativa and Healthy Controls. JAMA Dermatol..

[161] Naik H.B., Jo J.H., Paul M., Kong H.H. (2020). Skin Microbiota Perturbations Are Distinct and Disease Severity-Dependent in Hidradenitis Suppurativa. J. Investig. Dermatol..

[162] Guet-Revillet H., Coignard-Biehler H., Jais J.P., Quesne G., Frapy E., Poiree S., Le Guern A.S., Le Fleche-Mateos A., Hovnanian A., Consigny P.H. (2014). Bacterial pathogens associated with hidradenitis suppurativa, France. Emerg. Infect. Dis..

[163] Ring H.C., Sigsgaard V., Thorsen J., Fuursted K., Fabricius S., Saunte D.M., Jemec G.B. (2019). The microbiome of tunnels in hidradenitis suppurativa patients. J. Eur. Acad. Dermatol. Venereol..

[164] Naik H.B., Nassif A., Ramesh M.S., Schultz G., Piguet V., Alavi A., Lowes M.A. (2019). Are Bacteria Infectious Pathogens in Hidradenitis Suppurativa? Debate at the Symposium for Hidradenitis Suppurativa Advances Meeting, November 2017. J. Investig. Dermatol..

